# Winged forelimbs of the small theropod dinosaur *Caudipteryx* could have generated small aerodynamic forces during rapid terrestrial locomotion

**DOI:** 10.1038/s41598-018-35966-4

**Published:** 2018-12-14

**Authors:** Yaser Saffar Talori, Yun-Fei Liu, Jing-Shan Zhao, Corwin Sullivan, Jingmai K. O’Connor, Zhi-Heng Li

**Affiliations:** 1 Department of Mechanical Engineering, Tsinghua University, Beijing, 100084 P. R. China; 2 Department of Biological Sciences, University of Alberta, CW405 Biological Sciences Building, Edmonton, AB T6G 2E9 Canada; 3 Philip J. Currie Dinosaur Museum, Wembley, AB TS0 30H Canada; 4 Key Laboratory of Vertebrate Evolution and Human Origins, Institute of Vertebrate Paleontology and Paleoanthropology, Chinese Academy of Sciences, Beijing, 100044 P. R. China

## Abstract

Pennaceous feathers capable of forming aerodynamic surfaces are characteristic of Pennaraptora, the group comprising birds and their closest relatives among non-avian dinosaurs. However, members of the basal pennaraptoran lineage Oviraptorosauria were clearly flightless, and the function of pennaceous feathers on the forelimb in oviraptorosaurs is still uncertain. In the basal oviraptorosaur *Caudipteryx* both the skeleton and the plumage, which includes pennaceous feathers forming wing-like arrangements on the forelimbs, are well known. We used mathematical analyses, computer simulations and experiments on a robot *Caudipteryx* with realistic wing proportions to test whether the wings of *Caudipteryx* could have generated aerodynamic forces useful in rapid terrestrial locomotion. These various approaches show that, if both wings were held in a fixed and laterally extended position, they would have produced only small amounts of lift and drag. A partial simulation of flapping while running showed similarly limited aerodynamic force production. These results are consistent with the possibility that pennaceous feathers first evolved for a non-locomotor function such as display, but the effects of flapping and the possible contribution of the wings during manoeuvres such as braking and turning remain to be more fully investigated.

## Introduction

Pennaceous feathers comparable in their basic structure to modern avian contour feathers evidently represent an ancestral feature of Pennaraptora, the clade comprising avians as well as the closely related non-avian theropod dinosaur clades Dromaeosauridae, Troodontidae, Scansoriopterygidae and Oviraptorosauria (Fig. S1)^1,2^. True pennaceous feathers appear to have been secondarily lost in scansoriopterygids, as they are absent even in specimens of this group in which other feather types are preserved^3,4^, but are present throughout birds and in at least some members of the remaining pennaraptoran clades. However, the occurrence of pennaceous feathers in oviraptorosaurs poses an intriguing and arguably underappreciated problem. Oviraptorosaurs are characterized by relatively large body sizes (~2.5 kg and greater, compared to <1 kg for *Archaeopteryx*) and proportionately short forelimbs with wing-like feather sheets attaching to the hand, and were almost certainly non-volant^5^. The wing-like forelimb feathers are proportionately shorter than in potentially volant animals, but their sheet-like arrangement suggests the potential to produce small aerodynamic forces. Although pennaceous feathers have the obvious functional advantage over the filamentous feathers of non-pennaraptoran theropods of being capable of forming aerodynamic surfaces with potential utility in volant behaviours (flapping flight and/or gliding), it is clear that pennaceous feathers evolved first for some other function and were later exapted for flight. This raises the question of why oviraptorosaurs possessed pennaceous feathers on their forelimbs. If pennaceous feathers were not being used for volant behaviours in oviraptorosaurs, what function did they serve? Given that oviraptorosaurs are the most basal pennaraptoran theropods, the answer to this question might even reflect the functional role for which pennaceous feathers originally evolved. This original function was presumably one for which pennaceous feathers were better suited than the plesiomorphic filamentous feathers, or else the transition in feather morphology would not have occurred.

Pennaceous feathers undoubtedly provided some degree of insulation in oviraptorosaurs, but it is unlikely that they were more useful in this capacity than their filamentous counterparts, so selection pressure for improved insulation probably was not responsible for the feather transition. Some early pennaraptorans may have flapped their incipient wings as a means of maintaining balance while subduing prey^6^, but basal oviraptorosaurs were likely herbivorous^7^, which would rule out this type of behaviour. It is possible that oviraptorosaurian pennaceous feathers played a role in display^1^, a function for which sheets of feathers might plausibly have been better suited than filamentous plumage. A more readily testable alternative, however, is that the tail fan and wing-like forelimbs that were presented at least in basal oviraptorosaurs were used to generate small aerodynamic forces that were mechanically helpful in the context of terrestrial locomotion. This would also explain why even the oldest (Yanliao pennaraptorans) and most primitive (*Caudipteryx*) taxa known to possess pennaceous feathers consistently sport aerofoil-like surfaces on the forelimbs, hindlimbs, and/or tail. A variant of this hypothesis is that the forelimbs were used for wing-assisted incline running^8^, a behaviour exhibited by some juvenile birds today that involves using the wings to aid in running up slopes and clambering over obstacles. However, wing loading in basal oviraptorosaurs was likely too high for WAIR to have been feasible^5^, and we focus here on the more basic possibility that the wings might have produced aerodynamic forces large enough to contribute to terrestrial manoeuvrability, as apparently occurs in extant ostriches^9^.

**Figure 1:**
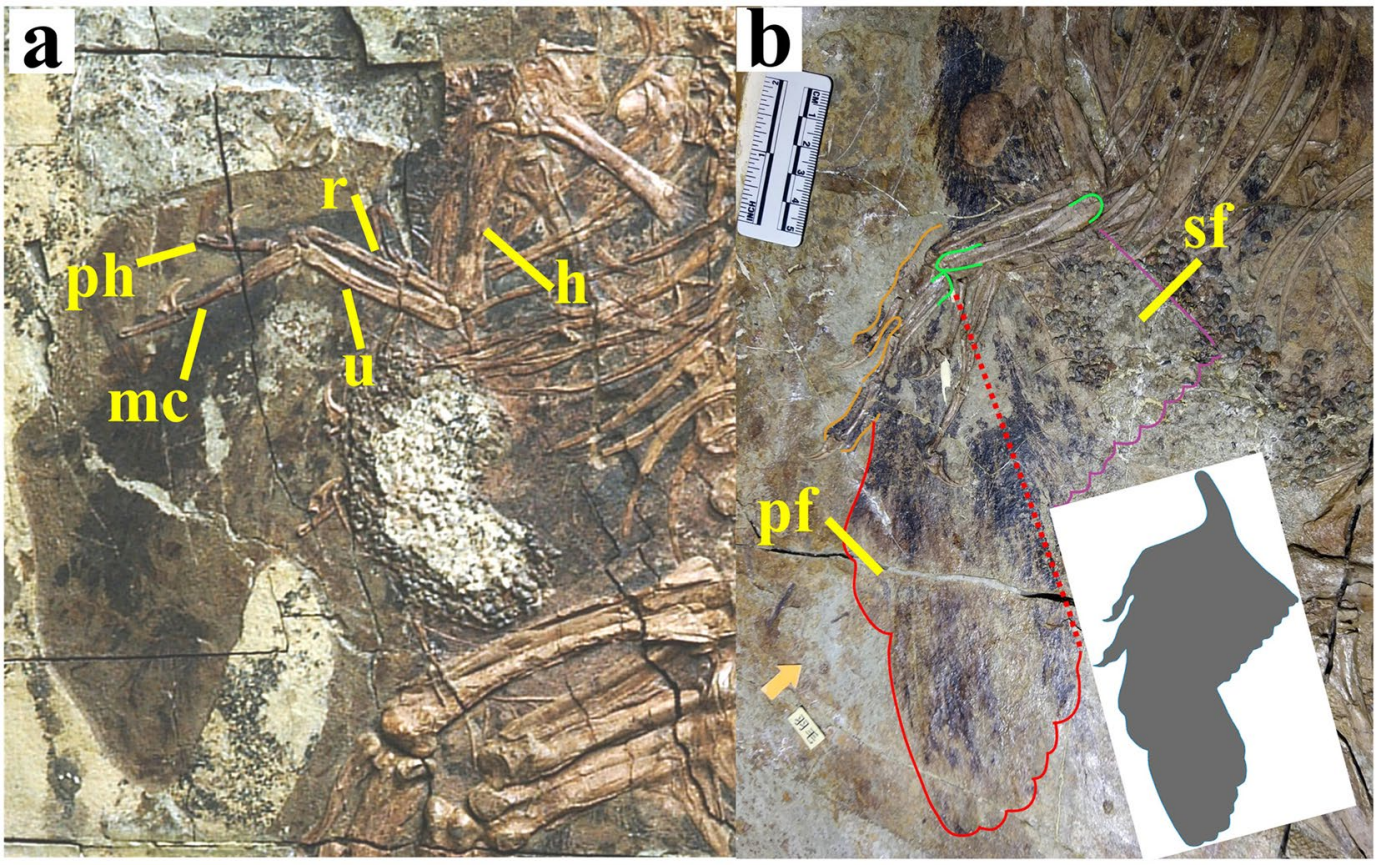
Fossils of *Caudipteryx* sp. IVPP V12430 (**a**) and *Caudipteryx dongi* IVPP V12344 (**b**), and inset showing outline of reconstructed *Caudipteryx* wing. Abbreviations: **h**, humerus; **mc**, metacarpal; **pf**, primary feathers; **ph**, phalanx; **r**, radius; **sf**, secondary feathers; **u**, ulna.

Among oviraptorosaurs, the plumage is partially known from fossil evidence in only three genera, namely *Protarchaeopteryx*^10^, *Caudipteryx*^10–12^ and *SimiliCaudipteryx*^13,14^. All three are basal oviraptorosaurs, falling outside the advanced clade Caenagnathoidea, and are relatively small dinosaurs, with body masses of ~2.5 kg for *Protarchaeopteryx*, ~5 kg for *Caudipteryx*, and ~4 kg for *SimiliCaudipteryx*^5^. Their status as basal forms within Oviraptorosauria strengthens the possibility that the function of the pennaceous feathers in these taxa may reflect their original adaptive role in Oviraptorosauria or even in Pennaraptora as a whole. Of the three genera, *Caudipteryx* (Fig. 1) is the best known; several specimens of the two species *C*. *dongi* and *C*. *zoui* having been described from the Lower Cretaceous Yixian Formation of northeast China^10–12^. Accordingly, we used a combination of mathematical analysis and physical modelling to assess the potential of the wings of *Caudipteryx* to produce small aerodynamic forces during terrestrial locomotion. The minor osteological differences between *C*. *dongi* and *C*. *zoui* would almost certainly not have affected the functionality of the wings, and so were disregarded in our study. We focused our analysis on a literally generic *Caudipteryx* with a body mass of 5 kg, a realistic value given that an empirical equation for estimating theropod body masses on the basis of femoral length^15^ produces results ranging from 4.74 kg to 5.18 kg (mean value = 4.96 kg) for a total of five described specimens^10–12^.

## Results

### Classical aerodynamic analysis of the wing of *Caudipteryx* during terrestrial running

**Figure 2:**
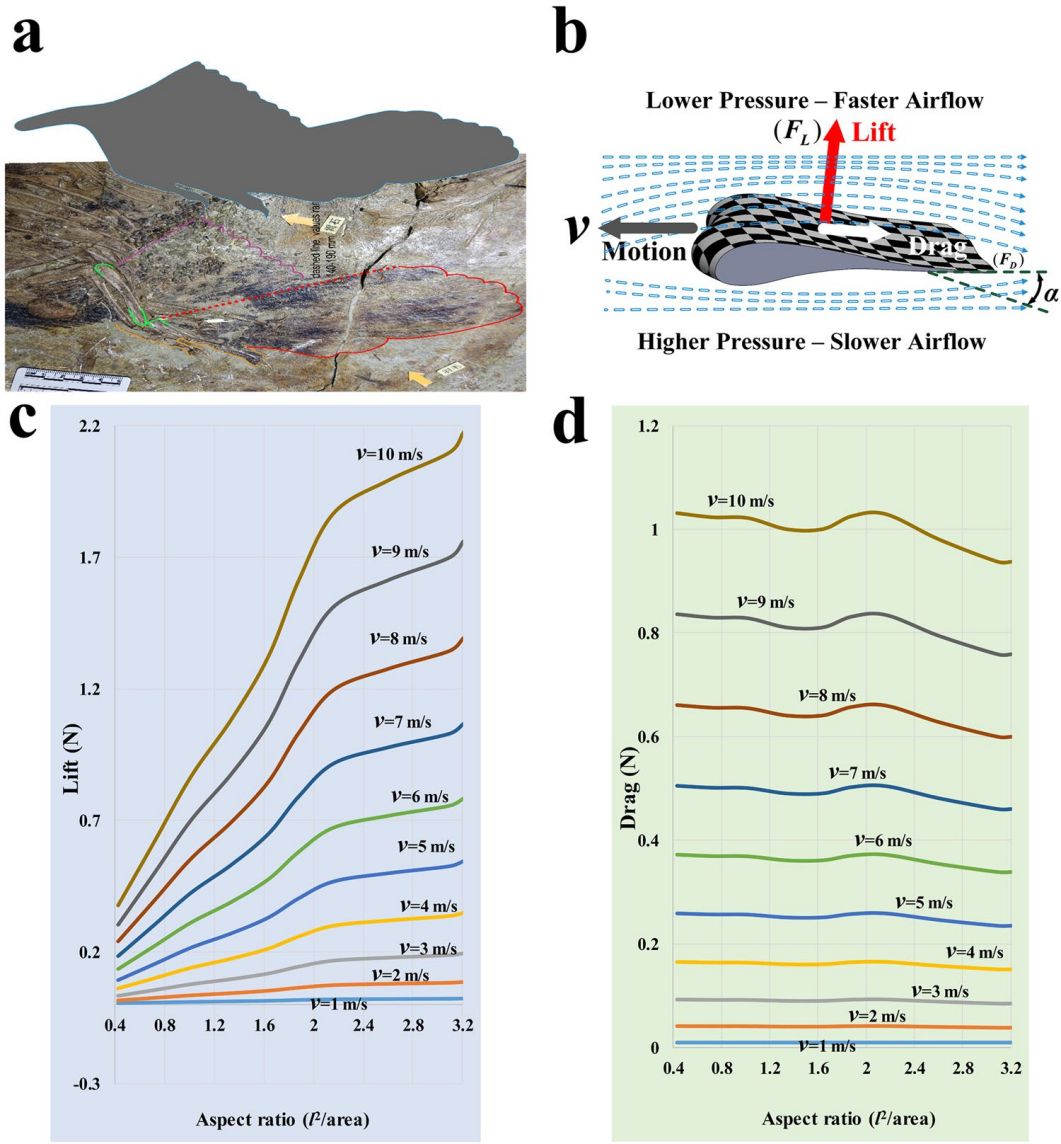
Classical analysis of the aerodynamic forces produced by the wings of *Caudipteryx* in terrestrial running on horizontal ground in still air. Reconstruction of wing outline (**a**) based on preserved plumage in fossils, and aerodynamic forces (**b**) arising from Bernoulli’s principle and interaction between incident horizontal airflow and wing held at optimal angle of attack. Aerodynamic equations estimate total lift force produced by both wings of *Caudipteryx* (**c**) and total drag force produced by both wings and the body (**d**), across a variety of running speeds and degrees of wing unfolding (reflected in the aspect ratio).

We initially used equations and assumptions from classical aerodynamics to estimate the lift and drag forces produced by the wings of *Caudipteryx* while running, representing the wing and its position in an essentially abstract manner. Values of total lift (the sum of the lift produced by both wings) and total drag (the sum of the drag affecting both wings, and the rest of the body) predicted using this approach for running speeds from 1 m/s to 10 m/s and aspect ratios from 0.4 to 3.2, reflecting different amounts of wing unfolding and therefore wing area, are shown in Fig. 2c,d. This analysis indicates that lift and drag would both have been considerably greater at higher running speeds, while higher aspect ratios (i.e. more complete unfolding of the wings) would have resulted in large increases in lift while having little effect on drag. Nevertheless, a *Caudipteryx* running at 8 m/s (estimated maximum running speed; see Materials and Methods) with wings fully extended would have experienced a total lift force of only ~1.4 N and a total drag force of only ~0.6 N, both very low in comparison to the estimated body weight of 49 N. Parameters and calculated results for this stage of analysis, assuming for the sake of example at a running speed of 8 m/s, are given in Table S1.

### Theoretical analysis of horizontal running with rectangular wings

**Figure 3:**
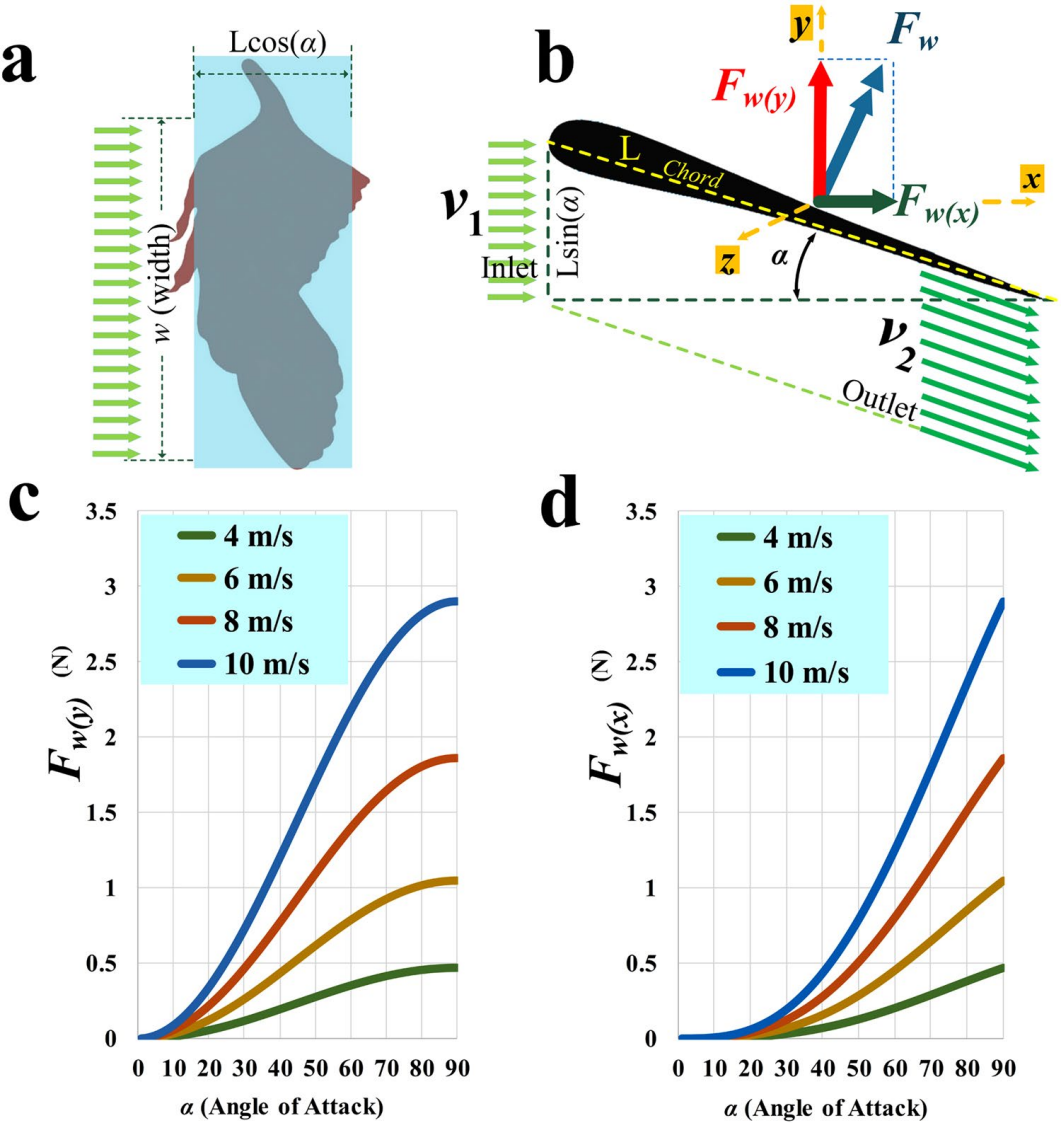
Aerodynamic analysis of the wing of *Caudipteryx* as a rectangular plate extending laterally from the body during horizontal running in still air. Dimensions of rectangle representing each wing (**a**), based on outline of left wing seen in dorsal view, and aerodynamic forces (**b**) arising from interaction of incident air with rectangular wing. Note that the cross-sectional shape of the wing has no effect on the aerodynamic forces produced in this particular analysis. Estimated vertical (**c**) and horizontal (**d**) forces for one rectangular wing, respectively approximating lift and drag, across a variety of running speeds and angles of attack.

In this stage of our analysis we represented the wing in a more concrete, albeit simplified, manner, as a thin, massless, rectangular plate that extended laterally from the body and was held at varying angles of attack (*α*) as the animal ran forward on level ground at constant speed in still air. The vertical and horizontal aerodynamic force components on the wing (*F*_*w*(*y*)_ and *F*_*w*(*x*)_, which respectively approximate lift and drag) are plotted as functions of angle of attack, across a range of airflow velocities, in Fig. 3c,d. Values of *F*_*w*(*y*)_ and *F*_*w*(*x*)_ both increase with angle of attack and with running velocity. Both forces are minimal at very low angles of attack, but *F*_*w*(*y*)_ begins to increase substantially as *α* rises to about 10° and *F*_*w*(*x*)_ begins to increase substantially as *α* rises to about 20°. For most *α* values, *F*_*w*(*y*)_ exceeds *F*_*w*(*x*)_ across all velocities, but at *α* = 90° *F*_*w*(*y*)_ and *F*_*w*(*x*)_ are approximately equal, ranging in magnitude from ~0.5 N (~1.0 N for both wings) at 4 m/s to ~1.8 N (~3.6 N for both wings) at 8 m/s.

Even at a velocity of 8 m/s, however, the estimated maximum for *Caudipteryx*, *F*_*w*(*y*)_ is <0.5 N for values of *α* below about 30° and *F*_*w*(*x*)_ is < 0.5 N for values of *α* below about 50°. Even at *α* = 90°, *F*_*w*(*y*)_ and *F*_*w*(*x*)_ both remain slightly under 2 N. At a moderate running speed of 4 m/s, *F*_*w*(*y*)_ approaches 0.5 N only when *α* = 80°, and *F*_*w*(*x*)_ approaches 0.5 N only at *α* = 90°. Although these calculations assume that airflow occurs only across the lower surface of the simple rectangular wing, simulation of airflow patterns (Fig. S2) shows that airflow across the upper surface creates stall (i.e. the air velocity becomes zero near the upper surface of the wing) at angles greater than about 45°. At angles above this threshold, *F*_*w*(*x*)_ greatly exceeds *F*_*w*(*y*)_, so that aerodynamic forces on the wing act predominantly in the horizontal direction. Furthermore, maximum airflow speed is about 16 m/s (twice the inlet speed of air initially encountering the wing) near the leading and trailing edges of the wing, which are now approximately dorsal and ventral in position (Fig. S2).

### Theoretical analysis of running at an angle of inclination with rectangular wings extended in a moving airstream

**Figure 4:**
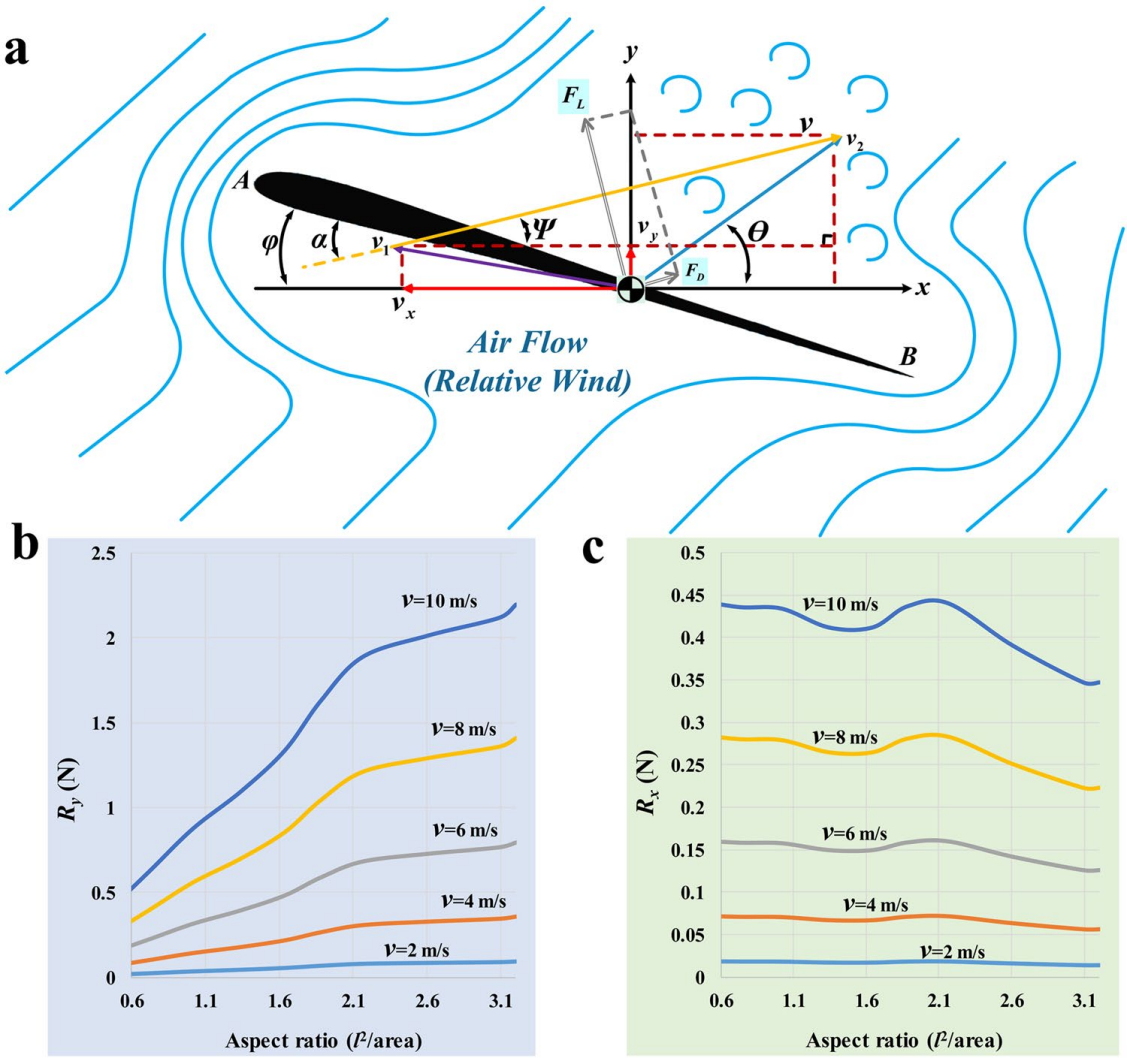
Aerodynamic analysis of the wing of *Caudipteryx* as a rectangular plate extending laterally from the body during running at an arbitrary angle of inclination in moving air. Aerodynamic forces (**a**) arising from interaction of incident air with rectangular wing. Estimated forces approximating lift (**b**) and drag (**c**) for both rectangular wings, across a variety of running speeds and degrees of wing unfolding (reflected in the aspect ratio).

In this stage of the analysis we returned to a more abstract representation of the wing, again utilizing equations from classical aerodynamics, but allowed the orientation of the wing, the orientation of the movement of *Caudipteryx*, and the orientation of the incident airstream to vary within a plane parasagittal to the animal’s body. The aerodynamic forces experienced by the wings are expressed by the components *R*_*x*_ and *R*_*y*_, which respectively approximate drag and lift. Figure 4b,c shows curves representing total *R*_*x*_ and *R*_*y*_ for the two wings as functions of aspect ratio, for a variety of running speeds but with an airstream moving at *v*_2_ = 0.5 m/s and at an angle of *θ* = 15° above the horizontal. The wings are assumed to be held in a horizontal position, and *Caudipteryx* is assumed to be running on level ground. At the lowest speeds considered (2 m/s and 4 m/s), *R*_*x*_ and *R*_*y*_ are both low, being respectively less than 0.1 N and less than 0.5 N. *R*_*x*_ tends to decrease slightly with aspect ratio, and *R*_*y*_ to increase slightly, whereas both *R*_*x*_ and *R*_*y*_ increase considerably at higher running speeds. The effect of aspect ratio on both *R*_*x*_ and *R*_*y*_ is more pronounced for high speeds than for low ones. By contrast, Fig. S3 shows *R*_*x*_ and *R*_*y*_ as functions of incident airflow angle (*θ*), with the wings fully unfolded (wing length 240 mm; aspect ratio ~3.2, calculated as the square of wing length divided by wing area). *R*_*x*_ decreases with airflow angle for all speeds considered, whereas *R*_*y*_ is unaffected by airflow angle (being nearly constant at around 0.2 N) at *v* = 2 m/s but decreases very slightly with airflow angle at higher speeds. Similarly, Fig. S4 shows *R*_*x*_ and *R*_*y*_ as functions of running speed for fully unfolded wings and *θ* = 15°. At low speeds *R*_*x*_ and *R*_*y*_ are both near zero. *R*_*y*_ increases rapidly with speed, reaching 1 N at about 6.7 m/s and 2 N at about 9.5 m/s, which would likely have somewhat exceeded the maximum running speed of a real *Caudipteryx*. *R*_*x*_, by contrast, increases more gradually, remaining under 0.5 N even at 10.0 m/s.

Because drag essentially represents a resistance force generated during locomotion, *Caudipteryx* would have had to expend metabolic energy in order to overcome drag. If we assume that the efficiency of the muscles involved in terrestrial locomotion was 95% and that about 10% of the power they produced was devoted to overcoming drag, the metabolic power output of the muscles would have had to increase from 1 to 12 W if running speed increased from 1 m/s to 10 m/s, as seen in Fig. S5. Total metabolic power would have increased sharply with velocity, but would have been no higher than about 6 J/s even at the maximum estimated running speed of 8 m/s. Wing aspect ratio would have had little effect on metabolic power expenditure.

### Analysis of forces generated by the wing in a series of positions corresponding to a downstroke

**Figure 5:**
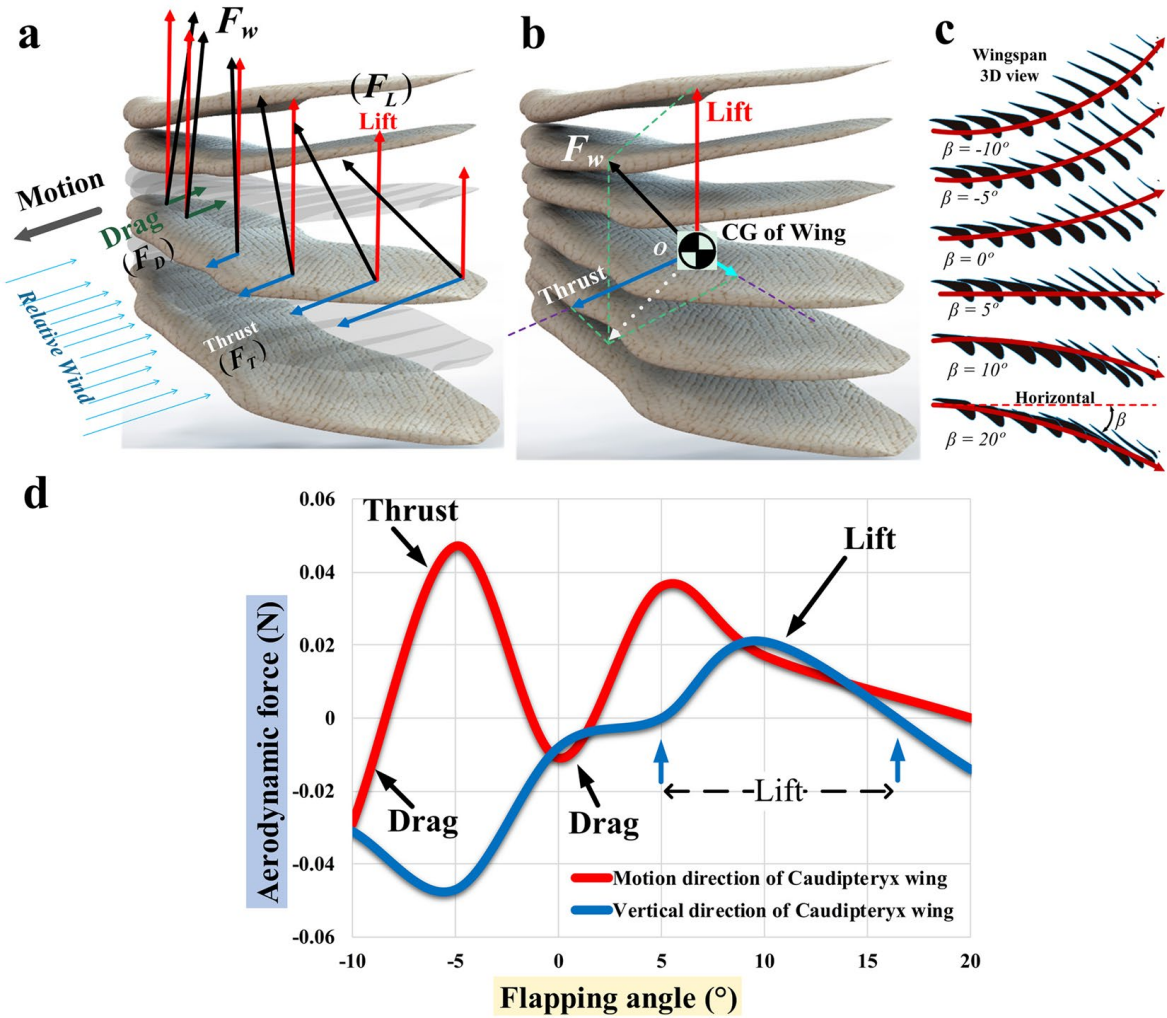
Aerodynamic analysis of the wing of *Caudipteryx* using a realistic 3D wing geometry and assuming the animal was running at 8 m/s on level ground in still air with the wing held in one of six positions corresponding to stages in a downstroke. Aerodynamic forces acting along the length of the wing at a flapping angle of *β* = 5° (**a**), with positions of maximum elevation (*β* = −10°) and maximum depression (*β* = 20°) also shown. Aerodynamic forces are averaged over the whole wing for a flapping angle of *β* = 5° and considered to be acting at the wing’s center of mass (**b**). Representation of the wing as a series of cross-sections for each of the six flapping angles considered (**c**), showing the twist along the length of the wing assumed for each flapping angle. Calculated vertical aerodynamic forces (positive or negative lift) and horizontal aerodynamic forces (thrust or drag) for a single wing, as a function of flapping angle (**d**).

In this portion of the analysis, we used the software package *ABAQUS* to estimate the airflow patterns and aerodynamic forces that would be produced if the realistically shaped wings of *Caudipteryx* were held fixed in one of several positions corresponding to stages in a downstroke during running on level ground. Each downstroke position was defined by a particular angle of elevation or depression of the wing at the shoulder joint (flapping angle, *β*), and the wing surface was assumed to twist along its length in a manner considered realistic for the downstroke position in question (Fig. 5).

**Figure 6:**
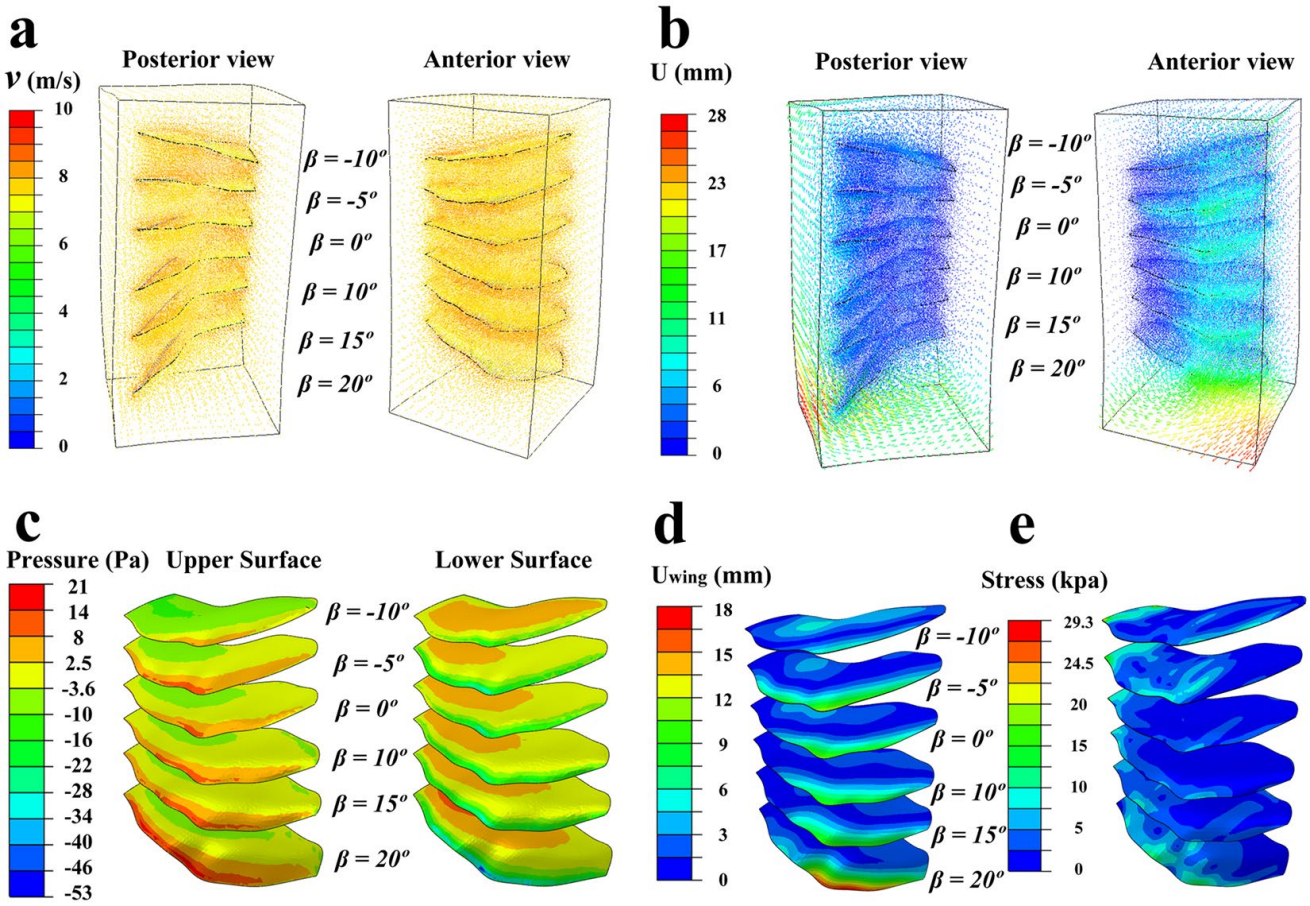
Further results of *ABAQUS* simulation of airflow about the wing of a running *Caudipteryx*, held fixed in various positions approximating stages of a downstroke. *Caudipteryx* is assumed to be running on level ground on still air. Velocity (**a**) and displacement (**b**) of the air passing the wing. Illustrations show pressure exerted by the air on the upper surface (left) and lower surface (right) of the wing. Lift results when pressure on the lower surface of the wing exceeds pressure on the upper surface. See also Supplementary Videos 1 and 2 (**c**). Displacement of (**d**) and stress (Von Mises) within (**e**) the substance of the wing. Displacement is concentrated near the leading edge of the wing, and bending and torsional stresses are concentrated on the shoulder joint and in the forelimb skeleton. For simplicity, the wing as a whole is assumed to have a Young’s modulus of 2.5 × 10^9^ Pascals and a Poisson’s ratio of 0.3^43^. See also Supplementary Videos 3 and 4.

The results of this procedure offer some preliminary insights into the likely aerodynamic effects of flapping while running, even though flapping was not explicitly modelled in our study. Assuming a horizontal incident airflow moving relative to the wing at a speed of *v* = 8 m/s, deviation of the airstream around the wing reached a maximum value of about 2.8 cm at a flapping angle of 20° (Fig. 6a,b), the maximal amount of depression considered in this part of the analysis (note that positive angles represent depression, while negative ones represent elevation). Lift is positive for flapping angles of 5° and 10°, in that pressure is greater on the lower surface of the wing than on the upper surface (Fig. 6c; Supplementary Videos 1 and 2). This pressure imposed by the airflow causes variable amounts of stress and deflection across the wing surface. The amount of deflection is maximal at *β* = 20°, with the leading edge of the wing rising by 1.8 cm (Fig. 6d and Supplementary Video 3). Furthermore, the wing base (i.e. the shoulder joint) and the forelimb skeleton experience considerable bending and torsional stresses (Fig. 6e and Supplementary Video 4). At all flapping angles tested, ranging from 20° to −10°, the aerodynamic force on the wing is resolvable into three components, namely lift acting in the upward direction, either thrust or drag acting parallel to the animal’s direction of motion, and a third force acting along the mediolateral axis of the wing. When directed laterally, the mediolateral force component could potentially help to maintain or bring about unfolding of the wing. Within the range of flapping angles tested, thrust is estimated to exceed drag for *β* = −8° to −1° and *β* = 2° to 20°. Lift is estimated to occur only at angles from 5° to 16°, which fall within the thrust-generating range. Thus, *Caudipteryx* could have produced both lift and thrust during fast running by unfolding its wings at modest angles of depression and appropriate angles of attack for producing lift (Fig. 5d). However, the aerodynamic forces on each wing would have been small, not exceeding 0.05 N in magnitude.

### Experiments with a *Caudipteryx* robot

**Figure 7:**
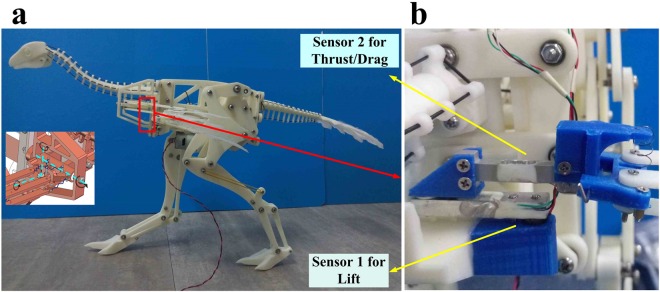
Robot based on skeletal proportions of *Caudipteryx* (**a**), with anatomically realistic wings. Close-up of sensors at base of wing (**b**), which were used to measure aerodynamic forces produced by the wings when the robot was placed in an airflow.

Airflow across the wings of the *Caudipteryx* robot (Fig. 7; Supplementary Video 5) at two different incident wind speeds created lift and drag (although not thrust), with the wings positioned at an angle of attack of *α* = 15–20°. At the lower wind speed of 3.5 m/s, the two wings produced a total of 0.32 N of lift and 0.15 N of drag, whereas at the higher wind speed of 6.0 m/s they produced 0.55 N of lift and 0.29 N of drag (Table 1).

**Table 1:** Lift and thrust/drag forces on the physically reconstructed, fully unfolded wings of a *Caudipteryx* robot, for incident airflow speeds of 3.5 m/s and 6 m/s.

Wind Speed (m/s)	Lift (N)			Thrust (N)		
	Right wing	Left wing	Total	Right wing	Left wing	Total
3.5	0.1585	0.1600	0.3185	−0.0791	−0.0708	−0.1499
6	0.2626	0.288	0.5506	−0.1422	−0.1510	−0.2932

## Discussion

Taken together, the results of various analyses and experiments carried out in this study consistently indicate that the potential of the wings of *Caudipteryx* to generate aerodynamic forces in the context of terrestrial running was small. This is true even when the aerodynamic forces produced are considered relative to the estimated body weight of 49 N (based on an estimated body mass of 5 kg). While the mathematical fixed-wing analyses varied somewhat in their results, all showed the total lift and drag experienced by the animal (or their near-equivalents) increasing as a function of running speed, but nevertheless having values under 4 N even at a running speed of 8 m/s, the estimated maximum for *Caudipteryx*. This value represents a small fraction (~8%) of the body weight of *Caudipteryx*. Similarly, the wings of the *Caudipteryx* robot constructed for this study produced a total lift of only 0.55 N and a total drag of only 0.29 N even at an incident windspeed of 6 m/s. Compared to lift and drag values generated for the same running speed (keeping in mind that the relevant parameter, with regard to the production of aerodynamic forces, is windspeed relative to the body) by the mathematical analyses, the experimental results indicate slightly lower lift and slightly greater drag. Nevertheless, the experiments and theoretical analyses broadly agree in indicating that the wings of a running *Caudipteryx* would not have been capable of generating substantial lift and drag. Production of thrust, as opposed to drag, was predicted only in the somewhat artificial *ABAQUS* simulation in which the wings were treated as being fixed in place but held in one of six positions approximating stages in a downstroke. In this scenario, the wings indeed generated thrust when they were considered to be held at moderate angles of elevation or depression, and twisted along their length to the degree that would be expected at points in a downstroke corresponding to these moderate flapping angles. However, the predicted amounts of thrust were so small (<0.05 N per wing) that they could have had no discernible effect on the animal’s movement.

These results are consistent with the conclusion reached by other studies^2,5^ that *Caudipteryx* was clearly non-volant. In order to glide rather than plummet after launching itself from an elevated perch, *Caudipteryx* would have had to produce lift equivalent to a substantial percentage of its own body weight. The results of our study clearly indicate that this would not have been possible, at least when the wings were held in fixed positions rather than actively flapped. Furthermore, our analysis suggests that the wings of *Caudipteryx* would have had negligible effects on its terrestrial locomotion even when they were held fully extended from the body in a symmetrical manner. Nevertheless, our study does not address the possibility that aerodynamic forces produced by the wings might have contributed to terrestrial manoeuvres such as turning, as apparently occurs in modern ostriches^9^, if the wings were deployed asymmetrically in some manner. Furthermore, we did not explicitly model flapping behaviour in our study. Although the results of our preliminary analysis of the downstroke suggest that the amounts of lift and thrust produced during flapping would have been minimal, the potential aerodynamic utility of flapping in *Caudipteryx* nevertheless requires further investigation. These issues will be considered in future studies using the same fundamental set of methods.

Our present results, however, are consistent with suggestions that pennaceous feathers and indeed wings originally evolved for a function other than production of aerodynamic forces, such as display. In the oviraptorosaur lineage, the modifications needed to transform the wings into effective aerodynamic structures may simply never have occurred. Among paravians, by contrast, pennaceous feathers formed the basis of a sophisticated aerodynamic apparatus permitting aerial locomotion in birds, the dromaeosaurid *Microraptor*^16^, and perhaps basal paravians in general^17^.

## Materials and Methods

The skeletal proportions we assumed for *Caudipteryx* were based primarily on BPM (Beipiao Paleontological Museum, Beipiao, China) 0001, a specimen with a complete, well-preserved skeleton but no associated plumage. The dimensions of the wing plumage were based primarily on measurements from IVPP (Institute of Vertebrate Paleontology and Paleoanthropology, Beijing, China) V12430 and V12344, specimens in which the forelimb feathers are relatively well preserved. The wing outline reconstructed in our study is shown in the inset to Fig. 1. We assumed the cross-sectional shape of the wing surface at various points between the shoulder joint and the wing tip to be similar to that seen in modern birds. The mediolateral length and the area of the reconstructed wing are respectively 0.24 m and 0.01797 m^2^, the aspect ratio of the fully unfolded wing is 0.32, and the average chord (i.e. anteroposterior width across the wing surface) of the wing is 0.10 m. Some parts of our analysis did not take the full three-dimensional structure of the reconstructed wing into account, but all parts were based on either the full reconstructed wing or some simplification of its geometry. In many cases aspect ratio was used as a proxy for the amount of wing unfolding, with larger aspect ratios corresponding to larger wing areas (because the wing was assumed to be more fully deployed).

We estimated the maximum running speed of *Caudipteryx* to be 8 m/s. This value was based on the skeletal hindlimb proportions of BPM 0001, and on adopting the assumptions used in Hutchinson (2004) with respect to the limb posture of small theropods and the range of Froude numbers (up to 17) they might have utilized in running. However, calculations in some parts of the analysis were carried out for running speeds of up to 10 m/s, in order to allow for the possibility that *Caudipteryx* could run somewhat faster than our method of estimation would indicate.

### Classical aerodynamic analysis of the wing of *Caudipteryx* during terrestrial running

In the first phase of our theoretical analysis of the wings of a running *Caudipteryx*, we used classical aerodynamic equations to estimate the forces the wings would have produced when extended laterally in a fixed position during running at constant speed on level ground in still air. Only the two-dimensional shape of each wing, as opposed to its cross-sectional area, affected these calculations. Parameters and calculated results for this part of the analysis, assuming for the sake of example that *Caudipteryx* was running at its estimated maximum speed of 8 m/s, are given in Table S1.

In this situation, each wing would generate lift and drag according to Bernoulli’s principle^8,18–27^ (Fig. 2a,b), which may be expressed as

1 $${p}+\frac{1}{2}\rho {{v}}^{2}={\rm{Constant}}$$

where *p* is pressure, *ρ* is the density of the air, and *v* is the velocity of the air relative to the wing, which in still air corresponds to the velocity of the running animal. The constant term in this equation represents the total strength possessed by the air making up a particular streamline (i.e. line of flow) across the wing. The lift and drag forces are calculated as

2 $$\{\begin{array}{c}{{F}}_{{L}}=\frac{1}{2}\rho {{S}}_{{p}}{{C}}_{{L}}{{v}}^{2}\\ {{F}}_{{D}}=\frac{1}{2}{\rho }{{S}}_{{p}}{{C}}_{{D}}{{v}}^{2}\end{array}$$

where *C*_*L*_ and *C*_*D*_ represent coefficients of lift and drag, respectively, *S*_*P*_ is the area of the wing projected into a plane perpendicular to the airflow (approximately half the true wing area, at an optimal angle of attack) and *v* represents airflow velocity. *C*_*L*_ and *C*_*D*_ cannot be precisely calculated based on the shape and material properties of a wing-like structure, but must generally be determined empirically or estimated based on reasonable assumptions^28–32^. For the wing of an extinct theropod, only the latter approach is applicable.

In the case of lift, we take *C*_*L*_ to be equal to 2.0, a conservative value for the maximal lift coefficient (i.e. the lift coefficient associated with an optimal angle of attack) of a slotted wing^19^. This is appropriate because the gaps between the ends of the individual remiges on the forelimb of *Caudipteryx* would act aerodynamically as slots.

Determining a realistic value for *C*_*D*_ is more complicated. The total drag experienced by each wing of *Caudipteryx* is the sum of induced drag, which occurs as a result of the same deflection of the airflow that produces lift, and profile drag, which results from a combination of friction with air in the laminar boundary layer passing over the wing surface and pressure exerted by air colliding with the wing as the latter moves forward. The coefficients associated with profile and induced drag, respectively *C*_*D(profile)*_ and *C*_*D(induced)*_, can be calculated as^19,32–34^

3 $$\{\begin{array}{c}{{C}}_{{D}({profile})}\approx \frac{{{S}}_{{w}}}{{{S}}_{{p}}}\frac{1.33}{{\mathrm{Re}}^{0.5}}\approx \frac{2.6}{{\mathrm{Re}}^{0.5}}\\ {{C}}_{{D}({induced})}\approx \frac{{k}{{C}}_{{L}}^{2}}{\pi {A}}\end{array}$$

where *S*_*w*_ is the area of the wing, *A* is the aspect ratio of the wing (*A* = 3.2, with the wing fully extended) and *k* is the induced drag factor, which is dependent on wing shape. Because the outline of the wing of *Caudipteryx* approximates an ellipse, we assign *k* a value of equal to π/4, appropriate for an elliptical wing. Re is the Reynolds number for the wing of *Caudipteryx*, representing the dimensionless ratio of inertial to viscous forces acting on the wing. Re may be calculated according to the standard formula Re = *ρvl*/*η* where *l* represents the length of the wing and *η* represents air viscosity. Assuming *ρ* = 1.21 kg/m^3^ and *η* = 1.8 × 10^−5^ Ns/m^2^, Re for the wing of *Caudipteryx* is 1.29 × 10^−5^, a value within the range for extant birds in flight. It is notable that *C*_*D(profile)*_ will decrease as velocity increases, and therefore Reynolds number increases, too. The total drag coefficient for one wing of *Caudipteryx* is^19,32–37^

4 $${C}_{D}\approx {C}_{D({profile})}+{C}_{D({induced})}\approx \frac{2.6}{{\mathrm{Re}}^{0.5}}+\frac{k{C}_{{L}}^{2}}{\pi A}$$

Total drag force on the wing, *F*_*D*_, represents the sum of profile drag force *F*_*D*(*profile*)_ and induced drag force *F*_*D*(*induced*)_, and may be written as

5 $$\{\begin{array}{c}{{F}}_{{D}({profile})}=\frac{1}{2}\rho {{S}}_{{p}}{{C}}_{{D}({profile})}{{v}}^{2}\Rightarrow {{F}}_{{D}({profile})}=\frac{1.33\rho {{S}}_{{p}}{{v}}^{2}}{{\mathrm{Re}}^{0.5}}\\ {{F}}_{{D}({induced})}=\frac{1}{2}\rho {{S}}_{{p}}{{C}}_{{D}({induced})}{{v}}^{2}\Rightarrow {{F}}_{{D}({induced})}=\frac{\rho {k}{{C}}_{{L}}^{2}{{S}}_{{p}}{{v}}^{2}}{2\pi {A}}\end{array}$$

6 $${{F}}_{{D}}={{F}}_{{D}({profile})}+{{F}}_{{D}({induced})}=\frac{1}{2}\rho {{S}}_{{p}}(\frac{2.6}{{\mathrm{Re}}^{0.5}}+\frac{{k}{{C}}_{{L}}^{2}}{\pi {A}}){{v}}^{2}$$

The total drag force on the wings and body of a moving *Caudipteryx* can then be expressed as^19^

7 $${{F}}_{D{,}\text{total}}=2[{{F}}_{{D}({profile})}+{{F}}_{{D}({induced})}]+{{F}}_{D{,}{body}}=\frac{1}{2}{\rho }{{v}}^{2}[2{{S}}_{{p}}(\frac{2.6}{{\mathrm{Re}}^{0.5}}+\frac{{k}{{C}}_{{L}}^{2}}{\pi {A}})+{{S}}_{{b}}{{C}}_{{D},{body}}]$$

where *S*_*b*_ is the area of the front of the body projected into a transverse plane and *C*_*D*(*body*)_ is the body drag coefficient. The term *S*_*b*_*C*_*D*(*body*)_ can be replaced by the area of a flat plate transverse to the air stream that would produce an equivalent amount of drag, or *A*_*e*_. Tucker^19^ deduced from experiments on living birds the formula *A*_*e*_ = (3.34 × 10^−3^)*m*^2/3^, where *m* is body mass (5 kg for *Caudipteryx*). Values of lift and total (i.e. wings and body) drag for different aspect ratio values from 0.4 to 3.2, reflecting different amounts of wing unfolding, are shown in Fig. 2c,d. Lift and drag curves in this figure were calculated for a range of running velocities, always assuming a headwind with an airspeed of 0.5 m/s and a lift coefficient of 2.0^38–42^.

### Theoretical analysis of a rectangular wing

In the next stage of our theoretical analysis, we modelled the interaction between the wing of *Caudipteryx* and the surrounding air in more concrete and physically explicit terms. We initially considered the wing of *Caudipteryx* to be a thin rectangular plate, which could be held extended laterally from the shoulder at varying angles of attack. The dimensions of the rectangle corresponded to the length and average chord of the real wing (0.24 m and 0.10 m respectively). We assumed the animal was running horizontally in a straight line at constant speed, with no air movement relative to the wing other than that produced by the forward motion of the body. We also ignored the effect of gravity on the wing. In the wing’s frame of reference, horizontally moving air would contact the lower surface of the wing and subsequently flow parallel to that surface, creating both vertical lift and horizontal drag (Fig. 3a,b).

The resultant of lift and drag, *F*_*w*_, would be a force vector directed backward and upward, acting perpendicular to the surface of the wing. *F*_*w*_ is given by

8 $${{\boldsymbol{F}}}_{{w}}=\mathop{{m}}\limits^{\bullet }({{v}}_{2}-{{v}}_{1})$$

where *m* is the mass of air flow across the underside of the wing (i.e. the total mass of air that flows across this surface per second) and $\mathop{{m}}\limits^{\bullet }=\frac{{dm}}{{dt}}$ is the derivative of mass with respect to time, and *v*_1_ and *v*_2_ respectively represent the velocity of incoming air as it contacts the leading edge of the wing and the velocity of outgoing air as it moves away from the trailing edge. The vector quantities *v*_1_ and *v*_2_ are equal in magnitude, but differ in direction because the airstream is reorientated as a result of contact with the wing. The mass flow is given by *m* = *ρBv*, indicating that mass flow is a function of the density of air (*ρ*_*air*_, in kg/m^3^), the area of the wing projected into a vertical plane (*B*, in m^2^), and velocity (*v*, in m/s, representing the common magnitude of *v*_1_ and *v*_2_). The vertical and horizontal components of *F*_*w*_, respectively approximating lift and drag, may be calculated according to:

9 $$\{\begin{array}{ccc}\sum {{F}}_{x} & = & 0\to {{F}}_{w(x)}+\mathop{{m}}\limits^{\bullet }({{v}}_{2(x)}-{{v}}_{1(x)})\\  & = & 0\to {{F}}_{w(x)}\\  & = & \mathop{{m}}\limits^{\bullet }({{v}}_{1(x)}-{{v}}_{2(x)})\to {{F}}_{w(x)}\\  & = & \mathop{{mv}}\limits^{\bullet }(1-{\rm{c}}{\rm{o}}{\rm{s}}\alpha )\\ \sum {{F}}_{y} & = & 0\Rightarrow {{F}}_{w(y)}+\mathop{{m}}\limits^{\bullet }({{v}}_{2(y)}-{{v}}_{1(y)})\\  & = & 0\to {{F}}_{w(y)}\\  & = & \mathop{{m}}\limits^{\bullet }({{v}}_{1(y)}-{{v}}_{2(y)})\to {{F}}_{w(y)}\\  & = & \mathop{{mv}}\limits^{\bullet }[0-(-{\rm{s}}{\rm{i}}{\rm{n}}\alpha )]\end{array}$$

where *α* is the angle of attack. Based on the expression given above for mass flow, the approximate vertical and horizontal aerodynamic forces on a hypothetical rectangular *Caudipteryx* wing are:

10 $$\{\begin{array}{c}{{F}}_{{w}({x})}=\rho {b}{{v}}^{2}(1-{\rm{c}}{\rm{o}}{\rm{s}}\alpha )\\ {{F}}_{{w}({y})}=\rho {b}{{v}}^{2}{\rm{s}}{\rm{i}}{\rm{n}}\alpha \end{array}$$

where *b* = *wl* sin*α* (m^2^), *l* = 0.100 m and *w* = 0.240 m.

In addition to this mathematical analysis, the fluid dynamics software package *ABAQUS* was used to simulate airflow patterns about the hypothetical flat rectangular wing, assuming the wing was held at various angles of attack during steady running at 8 m/s (Fig. S2).

### Theoretical analysis of running at an angle of inclination with wings extended in a moving airstream

In this stage of the analysis we again assumed the wing to be a rectangular plate (*AB* in Fig. 4a). We considered the wing to be moving at a velocity *v*_1_, reflecting the velocity of the body of *Caudipteryx* in running either across level ground or on a slope, and encountering air moving in a horizontal or inclined direction at a velocity *v*_2_. In this situation the relative velocity of the airflow with respect to the wing, *v*, is the resultant of *v*_1_ and *v*_2_: *v*_2_ = *v*_2_ – *v*_1_ (Fig. 4a). The wing is held at an angle *φ* relative to the horizontal, but this is not necessarily equivalent to the angle of attack *α* because the airstream may not be moving in a horizontal direction. Accordingly, the equations used in this stage of the analysis offer a general quantitative description of the wing of *Caudipteryx* interacting with an airstream, assuming only that the wing extends sideways from the body (rather than being elevated or depressed, though it may be rotated about its long axis) and that both *Caudipteryx* and the airstream are constrained to a two-dimensional plane. This approach could therefore be used to evaluate the ability of the wing to produce aerodynamic forces helpful in executing manoeuvres such as braking and turning, although we did not explicitly pursue the issue in this study.

The amplitude of the relative velocity ***v*** can be calculated based on the cosine law of triangles:

11 $${v}=\sqrt{{({{v}}_{{x}}+{{v}}_{2}{\rm{c}}{\rm{o}}{\rm{s}}\theta )}^{2}+{({{v}}_{2}{\rm{s}}{\rm{i}}{\rm{n}}\theta -{{v}}_{{y}})}^{2}}$$

where *v*_*x*_ and *v*_*y*_ represent the horizontal and vertical components of the absolute speed of the *Caudipteryx* wing (i.e. the horizontal and vertical components of *v*_1_), and *θ* is the angle of the airflow relative to the horizontal. According to the sine law of triangles:

12 $$\psi ={\rm{a}}{\rm{r}}{\rm{c}}{\rm{t}}{\rm{a}}{\rm{n}}(\frac{{{v}}_{2}{\rm{s}}{\rm{i}}{\rm{n}}\theta -{{v}}_{{y}}}{{{v}}_{2}{\rm{c}}{\rm{o}}{\rm{s}}\theta +{{v}}_{{x}}})$$

where *ψ* is the angle between ***v***, the vector of airflow relative to the wing, and the horizontal. The angle of attack *α*, representing the angle between an equivalent chord *AB* (i.e. a line extending between the leading and trailing edges of the wing, in the plane of the wing surface) and the relative airflow velocity *v* is

13 $$\alpha =\phi +\psi =\phi +\arctan (\frac{{{v}}_{2}\,\sin \,\theta -{{v}}_{y}}{{{v}}_{2}\,\cos \,\theta +{{v}}_{x}})$$

where *φ* is the angle between the horizontal and the equivalent chord *AB*. Based on aerodynamic theory, the lift and drag exerted on the wing are then

14 $$\{\begin{array}{c}{{F}}_{{L}}=\frac{1}{2}{{C}}_{{L}}\rho {s}{{v}}^{2}=\frac{1}{2}{{C}}_{{L}}\rho {s}[{({{v}}_{{x}}+{{v}}_{2}{\rm{c}}{\rm{o}}{\rm{s}}\theta )}^{2}+{({{v}}_{2}{\rm{s}}{\rm{i}}{\rm{n}}\theta -{{v}}_{{y}})}^{2}]\\ {{F}}_{{D}}=\frac{1}{2}{{C}}_{{D}}\rho {s}{{v}}^{2}=\frac{1}{2}{{C}}_{{D}}\rho {s}[{({{v}}_{{x}}+{{v}}_{2}{\rm{c}}{\rm{o}}{\rm{s}}\theta )}^{2}+{({{v}}_{2}{\rm{s}}{\rm{i}}{\rm{n}}\theta -{{v}}_{{y}})}^{2}]\end{array}$$

where *F*_*L*_ represents the magnitude of lift, acting perpendicular to the relative velocity *v*, and *F*_*D*_ represents the magnitude of drag, acting opposite to the relative velocity ***v*** (Fig. 4a). *C*_*L*_ and *C*_*D*_ are the lift and drag coefficients of the *Caudipteryx* wing, while *ρ* represents the density of the moving air and *s* is the effective area of the wing. Therefore, the resultant force ***R*** acting on the wing is given by

15 $$\begin{array}{c}{\boldsymbol{R}}=[\begin{array}{c}{{R}}_{{x}}({{v}}_{{x}}{,}{{v}}_{{y}}{,}{{v}}_{2})\\ {{R}}_{{y}}({{v}}_{{x}}{,}{{v}}_{{y}}{,}{{v}}_{2})\end{array}]=[\begin{array}{c}{{F}}_{{D}}\,{\rm{c}}{\rm{o}}{\rm{s}}\,\psi -{{F}}_{{L}}\,{\rm{s}}{\rm{i}}{\rm{n}}\,\psi \\ {{F}}_{{D}}\,{\rm{s}}{\rm{i}}{\rm{n}}\,\psi +{{F}}_{{L}}\,{\rm{c}}{\rm{o}}{\rm{s}}\,\psi \end{array}]\\ {\boldsymbol{R}}=[\begin{array}{c}\frac{1}{2}\rho {s}[{({{v}}_{{x}}+{{v}}_{2}{\rm{c}}{\rm{o}}{\rm{s}}\theta )}^{2}+{({{v}}_{2}{\rm{s}}{\rm{i}}{\rm{n}}\theta -{{v}}_{{y}})}^{2}]({{C}}_{{D}}\,{\rm{c}}{\rm{o}}{\rm{s}}\,\psi -{{C}}_{{L}}\,{\rm{s}}{\rm{i}}{\rm{n}}\,\psi )\\ \frac{1}{2}\rho {s}[{({{v}}_{{x}}+{v}_{2}{\rm{c}}{\rm{o}}{\rm{s}}\theta )}^{2}+{({{v}}_{2}{\rm{s}}{\rm{i}}{\rm{n}}\theta -{{v}}_{{y}})}^{2}]({{C}}_{{D}}\,{\rm{s}}{\rm{i}}{\rm{n}}\,\psi +{{C}}_{{L}}\,{\rm{c}}{\rm{o}}{\rm{s}}\,\psi )\end{array}]\end{array}$$

This equation indicates that resultant force is dependent on the magnitude of the relative airflow v, as well as on the angle of inclination of the airflow *θ* and the lift and drag coefficients (*C*_*L*_ and *C*_*D*_).

The total power (*P*_*horizontal*_) that the muscles of *Caudipteryx* must constantly supply in order to continue running at a given speed *v* is the dot product of *v* and the total drag on the animal, ***F***_*D*,*total*_:

16 $${{P}}_{{horizontal}}={{\boldsymbol{F}}}_{{D},{total}}\cdot {\boldsymbol{v}}=\frac{1}{2}\rho {{v}}^{3}[2{{S}}_{{p}}(\frac{2.6}{{\mathrm{Re}}^{0.5}}+\frac{{k}{{C}}_{L}^{2}}{\pi {A}})+{{A}}_{{e}}]$$

As was the case in the earlier stage of our analysis, ***F***_*D*,*total*_ represents the sum of the drag on both wings and on the body.

The input metabolic power needed for the muscles to generate *P*_*horizontal*_ would be *P*_*input*_ = *P*_*horizontal*_/η, where η represents the efficiency of the muscles of *Caudipteryx*. Assuming 10% of total muscular power would have been devoted to functions other than locomotion at any given time, a more realistic computation of input power would be

17 $${{P}}_{{input}}=[1.1{P}]/\eta $$

The total metabolic power (i.e. input metabolic power) expended by *Caudipteryx* during terrestrial running is shown, for a range of velocities, in Fig. S5. In performing these metabolic power calculations, we assumed values of zero both for *φ* and for *γ*, the angle between the horizontal and the velocity vector of *Caudipteryx v*_1_. This implies that *Caudipteryx* was running on a horizontal substrate, with the wing also held horizontally. The angle of attack would then depend on the angle of the incident airflow *v*_2_. In this situation the aspect ratio of the wing, which would effectively vary with the degree of wing folding, would have an effect on the horizontal and vertical components of the resultant force (*R*_*x*_ and *R*_*y*_), which approximately correspond to drag and lift for small values of *θ*.

### Analysis of forces generated by the wing in a series of positions corresponding to a downstroke

In order to produce aerodynamic thrust that might contribute to propulsion, a running *Caudipteryx* would need to flap its wings, not merely hold them extended in a fixed position relative to the body. Our analysis did not explicitly model flapping, but as a preliminary assessment of the aerodynamic forces associated with flapping wing movements we examined the effects of running with the wing held fixed in a series of positions approximating different stages in a downstroke (Fig. 5). A realistic 3D wing geometry was assumed in this part of the analysis, and *Caudipteryx* was considered to be running horizontally in still air at a speed of 8 m/s. Each downstroke position was defined by an angle of elevation or depression relative to the horizontal, which we term here the flapping angle *β*, and by twisting of the wing surface along its length, causing variation in the angle of attack across different parts of the wing. Twisting was modelled explicitly by dividing the wing along its length into a series of transversely narrow strips, with the angle of attack *α* changing incrementally from one strip to the next. The amount and direction of twisting were chosen, for each value of *β*, to produce a geometry corresponding to the shape the wing would be expected to adopt at that stage in a real downstroke. This part of analysis is thus physically unrealistic in that, during running with the wing held fixed at a particular *β* angle rather than actively moving up and down, no force would be available to produce the wing twisting that is being assumed to occur. However, we consider our attempt to model this situation to represent an informative thought as an experiment addressing the question of what forces the wing would be expected to produce if it could be held fixed with particular amounts of twisting at a range of *β* angle. Twisting of the wing makes possible the production of thrust, which acts in an opposite direction to drag. Accordingly, thrust and drag can be represented as a single thrust/drag vector acting on the wing, and pointing anteriorly if thrust exceeds drag but posteriorly if the opposite is the case.

We used the software package *ABAQUS* to estimate the aerodynamic forces associated with holding the wing fixed in each downstroke position. Because it is unlikely that *Caudipteryx* had a range of shoulder joint motion approaching that of modern birds, we considered only six modest flapping angles (*β* = −10°, −5°, 0°, 5°, 10° and 20°, with negative values corresponding to elevation and positive ones to depression) in this part of our analysis.

Figure 5a shows forces of lift and thrust/drag acting on the wing, in addition to their resultant force ***F***_*w*_, for various points between the shoulder and wingtip. When averaged over the entire wing (Fig. 5b), ***F***_*w*_ normally includes three components: lift, thrust/drag, and a force acting along the mediolateral length of the wing. The magnitude and direction of the averaged ***F***_*w*_, considered to act at the wing’s center of gravity, depend on the speed of the airstream in addition to wing 3D shape and wing position as expressed by flapping angle, angle of attack (at the base of the wing) and the lengthwise twisting of the wing’s surface. Drag tends to predominate over thrust near the base of the wing, but the reverse occurs near the tip, because the primary feathers play the main role in producing thrust (Fig. 5a).

Parameters simulated in this part of the analysis include velocity vectors for the airflow surrounding the wing (Fig. 6a,b); aerodynamic pressure over the top and bottom surfaces of the wing (Fig. 6c; Supplementary Videos 1 and 2); stresses on, as well as deflections of, the wing resulting from interaction with the airflow (Fig. 6d,e; Supplementary Videos 3 and 4); and lift and thrust/drag produced by the wing, shown as functions of the flapping angle (Fig. 5d). In Fig. 5d, interpolation based on results from the six discrete flapping angles tested in the analysis was used to produce continuous curves.

### Experiments with a *Caudipteryx* robot

We constructed a robot (see Supplementary 3D Model) based on the skeletal and plumage anatomy of fossil specimens of *Caudipteryx*. The skeletal proportions of the robot were based mainly on BPM 0001, whereas the proportions and arrangement of the remiges were based mainly on IVPP V12430 and IVPP V12344. Most of the robot was built from ABS plastic, but the wing plumage was composed of trimmed feathers taken from modern birds (Fig. 7a). Because there is no evidence for tertiary feathers in *Caudipteryx*, feathers were attached only to the hand and forearm segments of the wing skeleton, using metal pins. An airflow from the anterior direction, relative to the body orientation of the *Caudipteryx* robot, was provided at speeds of 3.5 m/s and 6.0 m/s (see Supplementary Video 5). Aerodynamic forces exerted on the wings, namely lift and thrust/drag, were measured using sensors positioned at the wing bases. The shoulder joint was designed to minimize inertial and frictional effects during the experiment.

## Electronic supplementary material

Supplementary Information

Supplementary Video 1

Supplementary Video 2

Supplementary Video 3

Supplementary Video 4

Supplementary Video 5

CAD model of skeleton of Caudipteryx robot

Dataset 1
