# Supplementary material for: Winged forelimbs of the small theropod dinosaur Caudipteryx could have generated small aerodynamic forces during rapid terrestrial locomotion
Source: Sci Rep. 2018 Dec 14;8:17854. doi: 10.1038/s41598-018-35966-4 (PMC6294793; doi:10.1038/s41598-018-35966-4)
Supplement: Supplementary file 1 — Supplementary Information [file 41598_2018_35966_MOESM1_ESM.pdf]

Supplementary Information: Winged forelimbs of the small theropod dinosaur *Caudipteryx* could have generated small aerodynamic forces during rapid terrestrial locomotion

Yaser Saffar Talori[1], Yun-Fei Liu[1], Jing-Shan Zhao[1*], Corwin Sullivan[2,3], Jingmai K. O'Connor[4], Zhi-Heng Li[4]

[1]Department of Mechanical Engineering, Tsinghua University, Beijing 100084, P. R. China.
[2]Department of Biological Sciences, University of Alberta, CW405 Biological Sciences Building, Edmonton, AB T6G 2E9, Canada.
[3]Philip J. Currie Dinosaur Museum, Wembley, AB TS0 30H, Canada.
[4]Key Laboratory of Vertebrate Evolution and Human Origins, Institute of Vertebrate Paleontology and Paleoanthropology, Chinese Academy of Sciences, Beijing, 100044, P. R. China.
*Correspondence and requests for materials should be addressed to Jing-Shan Zhao (jingshanzhao@mail.tsinghua.edu.cn).

Figures and Tables

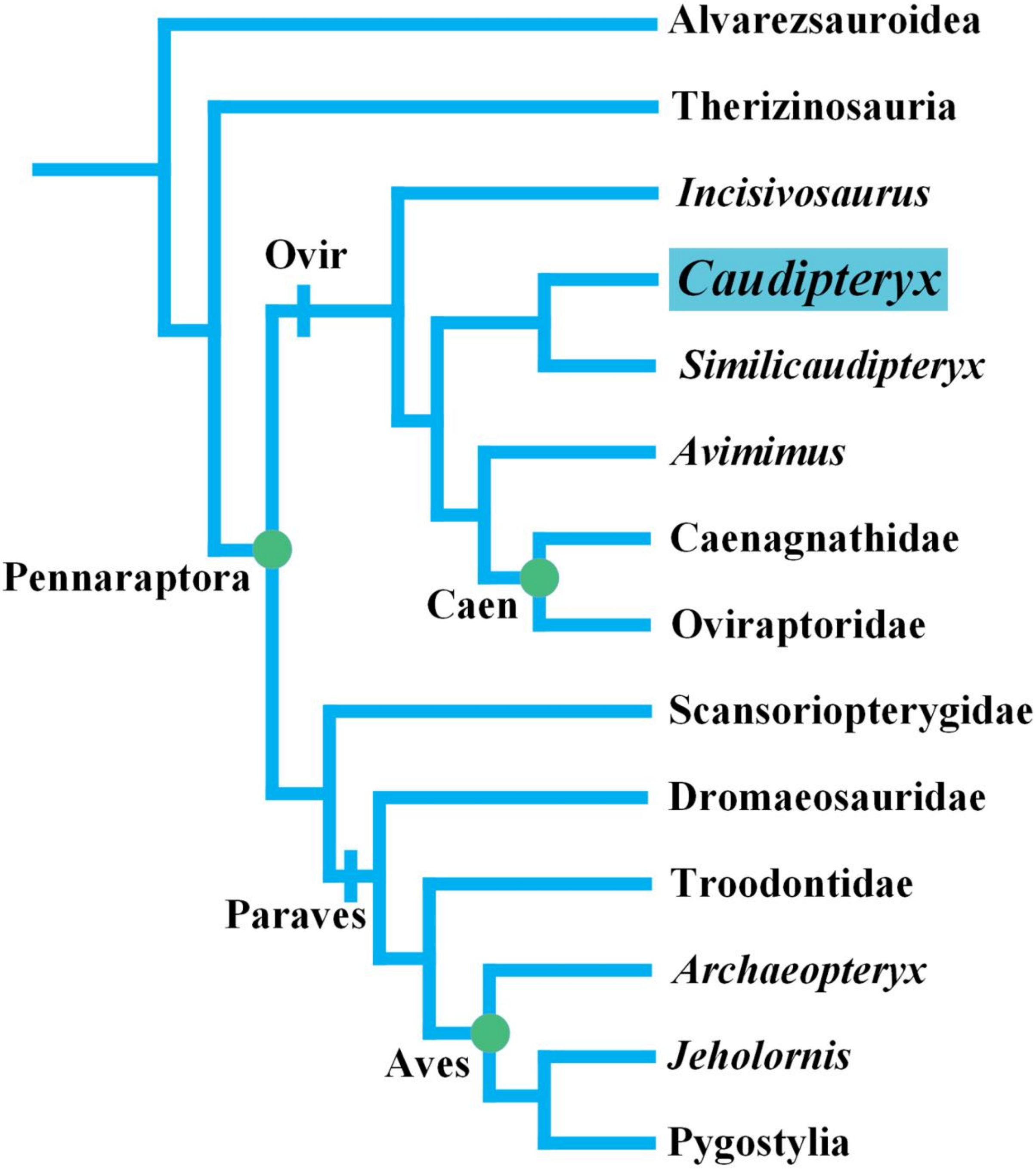

Figure S1. Cladogram showing the phylogenetic position of *Caudipteryx* among advanced theropod dinosaurs, based on an informal consensus of recent phylogenetic studies[1,4,45-47]. Abbreviations: Caen, Caenagnathoidea; Ovir, Oviraptorosauria.

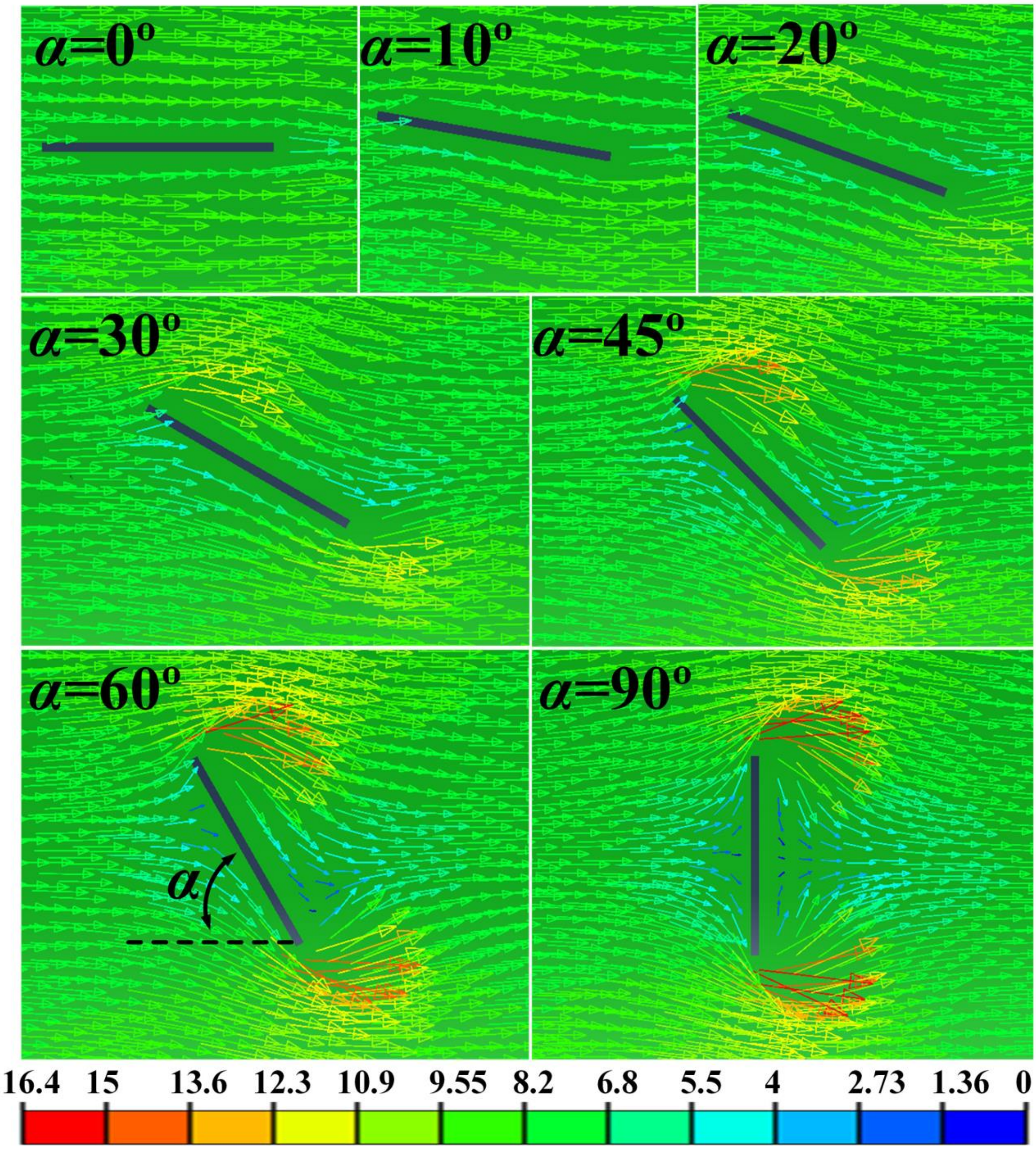

Figure S2. Simulated airflow patterns around a rectangular *Caudipteryx* wing held at varying angles of attack α during horizontal running at 8 m/s in still air. Separation of airflow from wing at angles of attack of 45° and greater indicates stalling. Colours represent velocity of airflow in m/s (see legend below main figure).

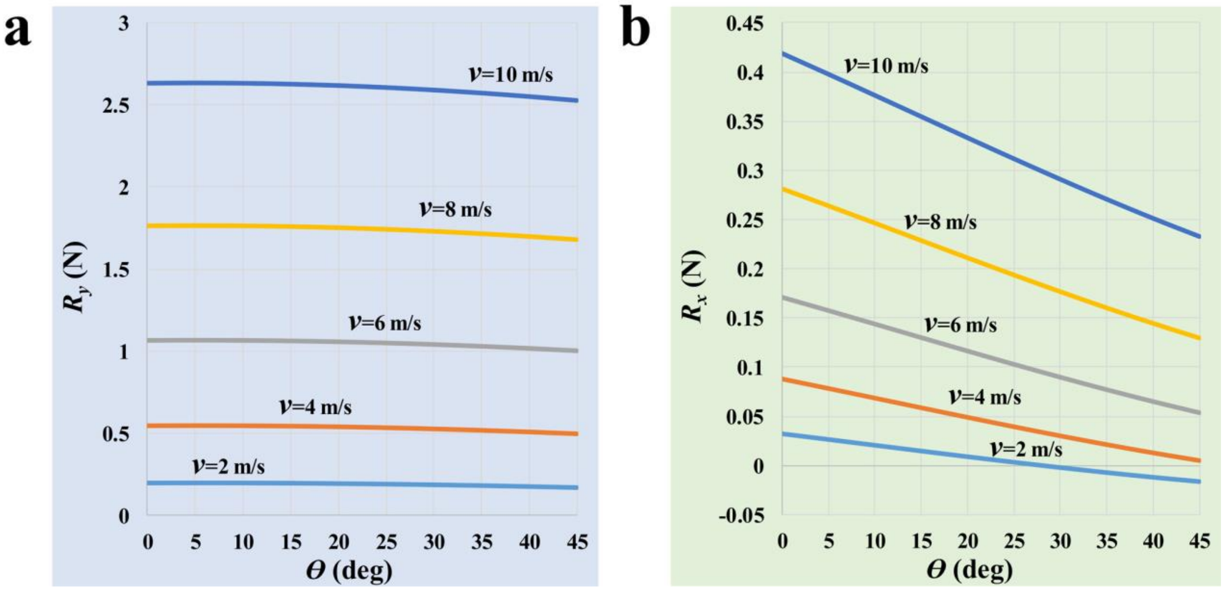

Figure S3. Further results from aerodynamic analysis of the wing of *Caudipteryx* as a rectangular plate extending laterally from the body during running in moving air. Total estimated forces approximating lift **(a)** and drag **(b)** for both rectangular wings, across a variety of running speeds and incident airflow angles (θ = inclination of incident airflow above the horizontal). Incident airflow speed is 0.05 m/s, and *Caudipteryx* is assumed to be running on level ground with the wings held horizontally.

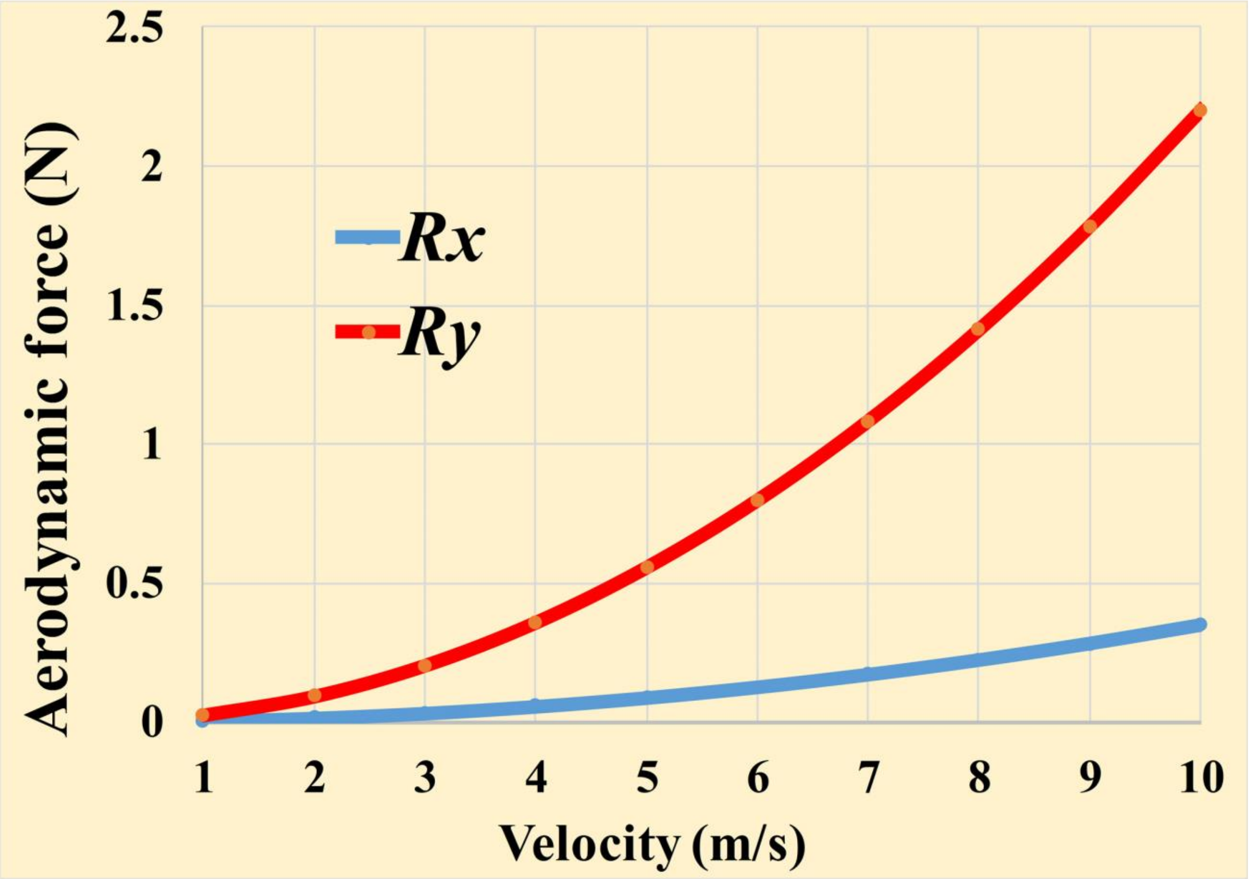

Figure S4. Further results from aerodynamic analysis of the wing of *Caudipteryx* as a rectangular plate extending laterally from the body during running in moving air. Total estimated forces approximating lift ($\boldsymbol{R}_y$) and drag ($\boldsymbol{R}_x$) for both rectangular wings as a function of running speed, assuming that the wings are fully unfolded (mediolateral length of each wing = 240 mm), the animal is running on level ground with the wings held horizontally, and the incident airflow is characterized by a speed of 0.05 m/s and an angle of 15° above the horizontal.

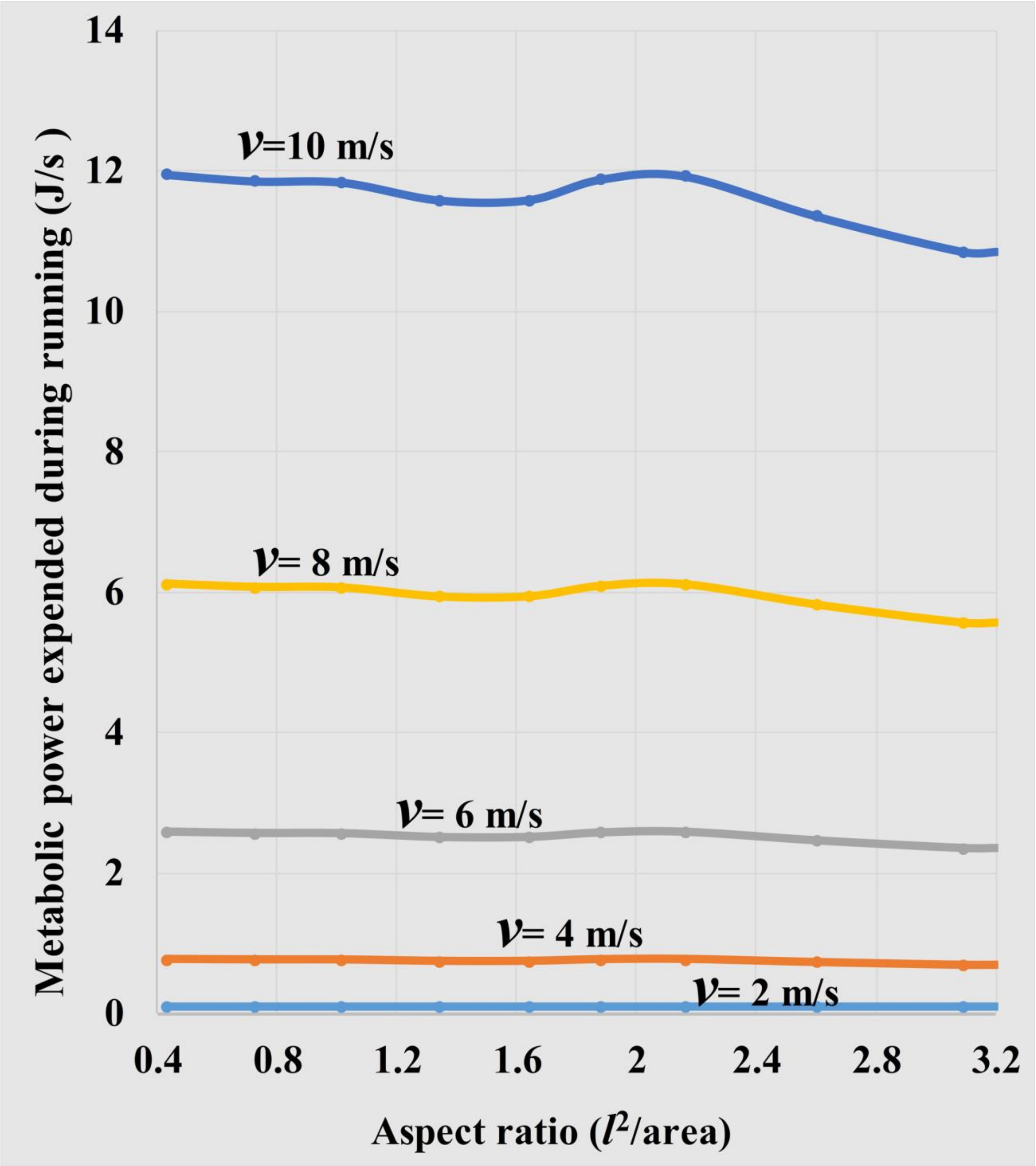

Figure S5. Metabolic power required to compensate for aerodynamic drag on the wings and body, for a *Caudipteryx* running at various speeds and with the wings extended to varying degrees. Efficiency (η) of the musculature involved in terrestrial locomotion was assumed to be 95%.

Table S1. Assumed and calculated parameters for classical aerodynamic analysis of *Caudipteryx* assuming a horizontal running speed of 8 m/s in still air, with the wings fully extended.

**Parameter**	**Value**	**Unit**
Viscosity of air (η_{air})	1.8×10^{-5}	Ns/m^2
Density of air (ρ_{air})	1.21	kg/m^3
Mass of single wing	0.500	Kg
Mediolateral length of single wing (l)	240	mm
Area of single wing	0.01797	m^2
Area of single wing when projected into plane perpendicular to airflow, at optimal angle of attack (S_p; roughly half of true wing area)	0.008986	m^2
Aspect ratio of single wing (l^2/area)	3.2	-
Speed of airflow relative to wings (v)	8	m/s
Reynolds Number of single wing ($\text{Re} = (\rho \boldsymbol{vl})/\eta$)	1.29×10^5	-
Induced drag factor (k, a function of wing shape)	π/4	-
Lift coefficient ($C_{L(\max)}$; accurate for $\alpha \approx 20^\circ$)	2	-
Area of flat plate transverse to airstream that would create drag equivalent to that of the body (A_e)	0.0097662	m^2
Drag coefficient ($C_D \approx C_{D(profile)} + C_{D(induced)} \approx 2.6/\text{Re}^{0.5} + kC_L^2/\pi A$)	0.319254	-
Wing loading ($N = F_L/S_p \approx mg/S_p$)	272.92	N/m^2
Lift produced by one wing ($F_L = 0.5\rho S_p C_L v^2$)	1.39176	N
Drag produced by one wing ($F_D = 0.5\rho S_p C_D v^2$)	0.2221	N
Total drag on wings and body ($F_{D,total} = 2[F_{D(profile)} + F_{D(induced)}] + F_{D,body}$)	0.60031	N
Lift to drag ratio (F_L/F_D) of single wing of *Caudipteryx*	6.26	-
Lift to drag ratio ($F_L/F_{D,total}$) for body and both wings	2.32	-

Supplementary References

45. Cau, A., Beyrand, V., Voeten, D. F. A. E., Fernandez, V., Tafforeau, P., Stein, K., Barsbold, R., Tsogtbaatar, K., Currie, P. J. & Godefroit, P. Synchrotron scanning reveals amphibious ecomorphology in a new clade of bird-like dinosaurs. *Nature*. **552**, 395–399 (2017).
46. Lamanna, M. C., Sues, H.-D., Schachner, E. R. & Lyson, T. R. A new large-bodied oviraptorosaurian theropod from the latest Cretaceous of western North America. *PLoS One*. **9**, e92022 (2014).
47. Wang, M., Stidham, T. A. & Zhou, Z. A new clade of basal Early Cretaceous pygostylian birds and developmental plasticity of the avian shoulder girdle. *Proceedings of the National Academy of Sciences of the United States of America*. **115**, 10708-10713 (2018).

Supplementary Video 1

Pressure (in Pascals) on the lower surface of the wing of *Caudipteryx*, for the six downstroke positions considered in the analysis. The wing is assumed to be held fixed and fully extended during horizontal running at 8 m/s in still air. The video shows the wing undergoing a transition from zero pressure to the maximum pressure associated with each downstroke position.

Supplementary Video 2

Pressure (in Pascals) on the upper surface of the wing of *Caudipteryx*, for the six downstroke positions considered in the analysis. The wing is assumed to be held fixed and fully extended during horizontal running at 8 m/s in still air. The video shows the wing undergoing a transition from zero pressure to the maximum pressure associated with each downstroke position.

Supplementary Video 3

Displacement (in metres) of the surface of the wing of *Caudipteryx*, for the six downstroke positions considered in the analysis. The wing is assumed to be held fixed and fully extended during horizontal running at 8 m/s in still air. The video shows the wing undergoing a transition from zero displacement to the maximum displacement associated with each downstroke position.

Supplementary Video 4

Stress (in Pascals) experienced by the wing of *Caudipteryx*, for the six downstroke positions considered in the analysis. The wing is assumed to be held fixed and fully extended during horizontal running at 8 m/s in still air. The video shows the wing undergoing a transition from zero stress to the maximum stress associated with each downstroke position.

Supplementary Video 5

Experiments with a *Caudipteryx* robot. Physical reconstruction of *Caudipteryx* in airflow produced by an electric fan, at wind speeds of 3.5 m/s and 6.0 m/s. Two force sensors were employed to measure the lift and drag produced by the wings under these conditions.

Supplementary 3D Model

CAD model of skeleton of *Caudipteryx* robot. Complete skeletal design used in constructing *Caudipteryx* robot, based primarily on osteology of BPM 0001.
